# Iodine nutritional status of pregnant women in an urban area of northern Taiwan in 2018

**DOI:** 10.1371/journal.pone.0233162

**Published:** 2020-05-15

**Authors:** Chun-Jui Huang, Chi-Lung Tseng, Harn-Shen Chen, Chii-Min Hwu, Kam-Tsun Tang, Justin Ging-Shing Won, Chiao-Wei Shih, Chang-Ching Yeh, Chen-Chang Yang, Fan-Fen Wang

**Affiliations:** 1 Division of Endocrinology and Metabolism, Department of Medicine, Taipei Veterans General Hospital, Taipei, Taiwan; 2 Faculty of Medicine, School of Medicine, National Yang-Ming University, Taipei, Taiwan; 3 Institute of Public Health, School of Medicine, National Yang-Ming University, Taipei, Taiwan; 4 Division of Gastroenterology, Department of Internal Medicine, Taoyuan General Hospital, Ministry of Health and Welfare, Taoyuan, Taiwan; 5 Institute of Clinical Medicine, National Yang-Ming University, Taipei, Taiwan; 6 Department of Obstetrics & Gynecology, Taipei Veterans General Hospital, Taipei, Taiwan; 7 Department of Obstetrics and Gynecology, Faculty of Medicine, School of Medicine, National Yang-Ming University, Taipei, Taiwan; 8 Institute of Environmental & Occupational Health Sciences, School of Medicine, National Yang-Ming University, Taipei, Taiwan; 9 Division of Clinical Toxicology & Occupational Medicine, Department of Medicine, Taipei Veterans General Hospital, Taipei, Taiwan; 10 Department of Medicine, Yangming Branch, Taipei City Hospital, Taipei, Taiwan; University of Mississippi Medical Center, UNITED STATES

## Abstract

Pregnant women are considered as one of the most vulnerable groups for iodine deficiency. The Nutrition and Health Survey in Taiwan 2013 revealed that the median urinary iodine concentration (UIC) of non-pregnant women of child-bearing age of 15–44 years was 124 μg/L, which was adequate in general, but insufficient according to pregnancy criteria. The aim of this study was to determine the iodine nutritional status of pregnant women in an urban area of Northern Taiwan. A hospital-based cross-sectional survey was conducted in Taipei Veterans General Hospital. Random spot urine samples were collected from January to October, 2018 and UIC was determined by inductively coupled plasma mass-spectrometry. A food frequency questionnaire was also delivered to the participants. The overall median UIC was 225.3 μg/L (IQR: 109.1–514.2 μg/L) for 257 pregnant women ranging from 21–47 years-old. The distribution of UIC was as follows: 35.4% with UIC <150 μg/L, 17.1% with UIC within 150–249 μg/L, 21.8% with UIC within 250–499 μg/L, and 25.7% with UIC ≥500 μg/L. The use of prenatal multivitamin was very common among the participants: 79.4% (n = 204) took multivitamin either every day or less frequently, with 52.5% (n = 135) taking one pill every day, and only 20.6% (n = 53) never took multivitamin during their pregnancy. Other commonly consumed iodine-containing foods were dairy products and fish. Our results indicate that the iodine status in the studied women is adequate. However, efforts are still needed to avoid iodine deficiency as well as iodine excess.

## Introduction

Iodine is a key component for the synthesis of thyroid hormone and is especially crucial in pregnancy and early life due to its influence on brain development of the fetus and infant. Iodine deficiency leads to a spectrum of disorders, namely iodine deficiency disorders, including cretinism, increased pregnancy loss and infant mortality, intellectual impairments, growth retardation, and thyroid dysfunction with or without goiter [1]. Cretinism and profound brain injuries are serious consequences of severe iodine deficiency during gestation [2]; however, even mild iodine deficiency in pregnancy can result in less favorable outcomes, impairing children’s cognition, intelligence quotient, and school performances [3–5]. Meta-analyses have revealed that the intelligence quotient of children living in iodine-deficient regions was 6.5 to 12.45 points lower than those residing in iodine-sufficient regions [6, 7]. In iodine-deficient regions, iodine supplementation to pregnant women has been shown to reduce cretinism, perinatal death, and infant mortality and improve the indices of maternal thyroid function [8–10].

Endemic goiter was the fifth prevalent disease in Taiwan in the 1940s [11]. In 1967, the Bureau of Salt Administration collaborated with the United Nations Children's Fund (UNICEF) to implement a universal salt iodization campaign by adding 33 ppm of potassium iodate to all salts. Four years later, the prevalence of goiter among school-age children decreased from 21.6% to 4.3%, showing that the iodine nutritional status of the Taiwanese population had improved from inadequate to adequate in 1971 [12]. However, after joining the World Trade Organization in 2002, the salt iodization policy was changed from mandatory to voluntary. The median urinary iodine concentration (UIC) of school-age children in the Nutrition and Health Survey in Taiwan (NAHSIT) in 2001–2002 was 123 μg/L, suggesting an adequate iodine status during the mandatory salt iodization period [13]. The median UIC of adults over 19 years old in NAHSIT in 2005–2008 was 100 μg/L, inferring that the iodine status was borderline adequate shortly after the policy change [14]. Ten years after implementing the voluntary salt iodization program, the median UIC of people aged over 6 years in NAHSIT in 2013 dropped to 96 μg/L, indicating mild iodine deficiency [15]. In recent years, the Taiwan government took several actions to improve iodine nutrition. First, campaigns promoting iodine-fortified salts were launched to increase public awareness and the Ministry of Health and Welfare mandated labeling of iodine content on all dietary salts. Second, the Taiwan Food and Drug Administration revised the Standards for Specification, Scope, Application, and Limitation of Food Additives to raise the iodine content in salt fortified with potassium iodide or potassium iodate from 12–20 mg/kg to 20–33 mg/kg in June, 2017 [16].

Due to increased urinary iodine excretion during pregnancy and the fetal dependency on maternal iodine intake for thyroid hormone synthesis, the recommended daily iodine intake suggested by the World Health Organization (WHO) in pregnancy is 250 μg per day, higher than the usual recommended daily iodine intake of 150 μg for adults [17]. In a population sample of pregnant women, adequate iodine nutrition, as classified by the WHO/UNICEF/Iodine Global Network (IGN), is defined when the median UIC is between 150–249μg/L, which is higher than the 100–199μg/L range for the adequacy in the general population [17]. Therefore, the median UIC of 124 μg/L for non-pregnant women of child-bearing age of 19–44 years found in NAHSIT 2013 may be sufficient in general, but is inadequate for pregnant women [15]. To date, there has not been any official publication on data of iodine nutritional status in pregnant women in Taiwan. This study thus aimed to determine the iodine nutritional status of pregnant women in an urban area of Northern Taiwan through a cross-sectional hospital-based survey. Additionally, dietary sources of iodine nutrition were investigated.

## Materials and methods

### Ethics statement

Informed consent was provided by all participants prior to their participation in the study. Ethical approval for this study was granted by the Institutional Review Board at TVGH (VGH IRB No: 2016-03-013A).

### Study design

This is a cross-sectional hospital-based survey that collects data on the iodine status of pregnant women in an urban area in Northern Taiwan. Only women aged 20 years or above who were confirmed pregnant and who visited the Obstetrics and Gynecology Outpatient Department of Taipei Veterans General Hospital (TVGH) for routine pregnancy checkups were included. Taipei Veterans General Hospital is a tertiary referral center that provides interprofessional care to pregnant women and their babies. Pregnant women generally visit Taipei Veterans General Hospital to receive prenatal checkups when they decide to deliver their babies at the hospital, which can occur in any trimesters of their pregnancy. The participants were recruited on a volunteer basis during their first prenatal checkup and were asked to provide a random spot urine sample and to complete a simple Food Frequency Questionnaire (FFQ). The women were approached at the nursing station where they received their routine checkups for blood pressure, heart rate, and urine dipstick test for proteinuria. If a woman had just urinated and could not provide another urine sample at their first visit, she could choose to provide her urine sample at follow-up visits. If a woman had provided a second urine sample on subsequent visits, only the first urine sample was analyzed.

### Urinary iodine analysis

Urine samples were collected from January to October, 2018. After the participants urinated in a cup, aliquots of urine were immediately transferred to 10-mL frozen tubes. Urine samples were stored at -20°C and the samples were subsequently transferred to 4°C environment one day before the analysis.

UIC was determined using inductively coupled plasma mass-spectrometry (ICP/MS) [18, 19]. Briefly, 0.3 mL of urine sample was added to 1 mL of internal standard (tellurium 500 μg/L) and then mixed with 4 mL of 2% ammonia solution (NH_3_∙H_2_O) and 0.05% of Triton X-100 in water solvent. With calibration, the controls and specimen were all transferred to tubes appropriate for the analysis using the Agilent 7700 Series ICP/MS. Samples were analyzed against a urine calibration curve (standard curve) to measure the unknown iodine concentration (I^127^) in the collected urine samples. The limit of detection was 1 μg/L. Samples with readings >1000 μg/L were diluted with water to fit into the calibration curve. All samples were automatically analyzed in triplicate immediately and the mean value was calculated. Quality control samples from the Ensuring the Quality of Urinary Iodine Procedures (EQUIP) program were also tested in each run of ICP/MS analysis with accurate results. To ensure validity of the assay, samples were also measured by a modified microplate method using ammonium persulfate as the oxidizing agent as previously described [13–15, 20–23]. There were no significant differences in the results obtained by the ICP/MS and the microplate method. Moreover, a “Successful” participation certificate of the EQUIP program was issued by the Centers for Disease Control and Prevention of the United States for the ICP/MS method in 2018.

### Food frequency questionnaire

The format of the FFQ was similar to what had been described previously [23] (S1 Appendix). The first part of the questionnaire contained five questions designed to survey the frequency of consumption of iodine-containing foods. The questions were asked in the following way: “How many days a week do you eat a certain type of food during gestation?” The types of food included were as follows: (1) seaweed (kelp or laver), (2) fish, (3) seafood (except fish), (4) dairy product (milk, cheese, yogurt, cream), and (5) prenatal multivitamin. The participants were asked to select the closest response item, whether it was 1, 3, 5, 7days per week, or never. The second part was designed to evaluate the use of iodized salt in the participant’s households. It began with the question of asking the frequency (days in a week) of eating outside. Then, the participants were asked about the kind of salt (iodized or non-iodized) used at their home. If the participants did not know the correct response, the item of “I don’t know” could be selected. For further analysis, the frequency of prenatal multivitamin intake was grouped into three categories: “always” (intake of prenatal multivitamin every day [i.e., 7 days per week]), “some” (intake of prenatal multivitamin 1, 3, or 5 days per week), and never. To evaluate the relationship between food consumption frequency and UIC, three categories of food intake were designated: “low intake” (intake less than or equal to one day per week), “medium intake” (intake equals to three days per week), and “high intake” (intake more than or equal to 5 days per week).

### Statistical analysis

The iodine nutritional status was expressed as the median UIC with inter-quartile range (IQR) for the sampled women. The following criteria were then employed in classifying the iodine nutrition of a population of pregnant women according to the WHO/UNICEF/IGN: median UIC <150 μg/L as iodine insufficient, 150–249 μg/L as adequate iodine nutrition, 250–499 μg/L as above the requirement, and ≥500 μg/L as excessive [17].

Statistical analysis was performed using the Statistical Package for the Social Sciences (SPSS) software, version 18.0. We used the Kolmogorov-Smirnov test to assess the normality of variables before performing further statistical analysis. Because UICs were not normally distributed, analysis of differences between the medians of UIC was performed with non-parametric tests, including the Mann–Whitney U test for two groups comparisons and the Kruskal–Wallis test with Dunn’s post hoc tests and Bonferroni correction for three groups comparisons. Pearson’s Chi-square test was used for comparisons between categorical variables. A two-tailed *p* value of less than 0.05 was considered as statistically significant.

## Results

A total of 257 pregnant women with a mean age of 33.9 (S.D. 4.3, range: 21–47) years were analyzed. Among the participants, 16 women were in their first trimester (gestation week 1–13+6), 86 in their second trimester (gestation week 14–27+6), and 155 in their third trimester (gestation week 28 to delivery), respectively.

### Urinary iodine concentration

The median UIC for the studied women was 225.3 μg/L (IQR: 109.1–514.2). The median UIC ranged from 222.8 to 230.8 μg/L in different age groups, from 214.9 to 259.7 μg/L in different residing regions, and from 223.3 to 269.0 μg/L in different trimesters. No statistically significant difference in UIC was observed between women of different age groups, residing regions, and trimesters (Table 1).

**Table 1:** Urinary iodine concentration (μg/L) and its distribution according to WHO cut-points for population sufficiency (3rd edition of WHO manual) [17].

	Sample (n)	Urinary iodine concentration (μg/L)						
	-	Median (IQR)	*p*-Value	<150 (%)	150–249 (%)	250–499 (%)	≧500 (%)	*p*-Value
Total	257	225.3 (109.1–514.2)		91 (35.4)	44 (17.1)	56 (21.8)	66 (25.7)	
**Age** (years)			0.95					0.90
20–29	43	230.8 (100.7–643.7)		13 (30.2)	10 (23.3)	9 (20.9)	11 (25.6)	
30–34	111	222.8 (128.4–534.5)		39 (35.1)	19 (17.1)	23 (20.7)	30 (27.0)	
35–47	103	225.3 (98.0–488.3)		39 (37.9)	15 (14.6)	24 (23.3)	25 (24.3)	
**Residing region**			0.23					0.42
Metropolis	168	214.9 (98.7–515.8)		65 (38.7)	27 (16.1)	33 (19.6)	43 (25.6)	
Suburb	89	259.7 (131.2–518.3)		26 (29.2)	17 (19.1)	23 (25.8)	23 (25.8)	
**Trimester**			0.51					0.32
First	16	269.0 (146.3–777.8)		5 (31.3)	2 (12.5)	4 (25.0)	5 (31.3)	
Second	86	229.0 (96.4–620.0)		31 (36.0)	12 (14.0)	15 (17.4)	28 (32.6)	
Third	155	223.3 (114.1–460.0)		51 (32.9)	31 (20.0)	37 (23.9)	36 (23.2)	

IQR: Interquartile range (25^th^ and 75^th^ percentiles

The distribution of urinary iodine concentration is as follow (Table 1): the overall percentage of pregnant women with a UIC <150 μg/L was 35.4%, and 17.1% of the studied women had a UIC within 150–249 μg/L. 21.8% of pregnant women had a UIC between 250 and 499 μg/L, while 25.7% of the studied women had a UIC ≥500 μg/L. A similar pattern of distribution of UIC was found in pregnant women of different age groups, regions of residence, and different trimesters (Table 1).

### Food frequency questionnaire

The most commonly consumed iodine-containing foods in the studied women were dairy products, with 81.7% of women indicating they ate these products on 3 or more days per week. Consumption of fish and seafood was not uncommon, but seaweeds were seldomly eaten by pregnant women. Among the studied women, 79.4% (n = 204) took multivitamin either every day or less frequently, with 52.5% (n = 135) taking one pill every day, and only 20.6% (n = 53) never took multivitamin). When the participants were asked about their household use of iodized salt, 42.4% (n = 109) reported that they were unaware of the type of salt (iodized or not) used at home. Notably, 41.3% (n = 106) said the household salt was iodized, and 16.3% (n = 42) reported the use of non-iodized salt. We also found that 55.3% of the participants had eaten out more than 5 days per week, and 26.5% of the studied population hardly ate home-prepared foods (Table 2).

**Table 2:** Result of food frequency questionnaire: Number of days per week in consuming various foods.

Food type	7 days (%)	5 days (%)	3 days (%)	1 day (%)	0 (%)	Median (IQR)
Seaweeds	3.5	2.3	26.5	47.1	20.6	1 (1–3)
Fish	6.2	12.8	42.0	29.6	9.3	3 (1–3)
Seafood (other than fish)	4.7	5.1	33.9	43.2	13.2	1 (1–3)
Dairy products	31.1	27.2	23.3	14.8	3.5	5 (3–7)
Prenatal multivitamin	52.5	12.5	6.6	7.8	20.6	7 (1–7)
Eat out	26.5	28.8	32.7	10.9	1.2	5 (3–7)

IQR: Interquartile range (25^th^ and 75^th^ percentiles)

### Prenatal multivitamin intake

There was a tendency for higher median UIC in the groups with more prenatal multivitamin intake, but it was not statistically significant (UIC 192.7, 241.5, and 242.6 μg/L for the “never”, “some”, and “always” groups, respectively; *p* = 0.70). The percentage of pregnant women with UIC ≥500 μg/L was also not significantly higher in the groups with prenatal multivitamin intake. When age was analyzed as a potential determinant of prenatal multivitamin intake, statistically significant difference (*p* = 0.02) was found, with the groups “always” and “some” having a slightly higher age (mean age, 33.9 ± 4.1 years old and 34.8 ± 4.2 years old, respectively) compared to that of the group “never” (mean age, 32.7 ± 4.4 years old) (Table 3).

**Table 3:** Group comparison according to the consumption of prenatal multivitamin.

Prenatal Multivitamin Intake	Never (n = 53)	Some (n = 69)	Always (n = 135)	*p*-Value
Age (S.D.)	32.7 (4.4)	34.8 (4.2)	33.9 (4.1)	0.02
UIC (IQR)	192.7 (107.3–481.9)	241.5 (107.0–530.3)	242.6 (109.0–534.5)	0.70
UIC≧500 μg/L (%)	11 (20.8)	19 (27.5)	36 (26.7)	0.65
**Residing area**				
Metropolis (%)	36 (67.9)	43 (62.3)	89 (65.9)	0.80
Suburb (%)	17 (32.1)	26 (37.7)	46 (34.1)	
**Trimesters**				
Early trimester (%)	21 (39.6)	23 (33.3)	58 (43.0)	0.41
Late trimester (%)	32 (60.4)	46 (66.7)	77 (57.0)	

Early trimester includes the first and second trimester, and late trimester is the thirst trimester.

*Kruskal–Wallis test was used to analyze age and median UIC. The other variables were analyzed using Pearson’s Chi-square test.

### UIC and food frequency

The relationship between median UIC and the consumption frequency of various food groups is as follow: statistically significant difference was observed between different consumption frequencies of seafood (UIC, 187.8, 280.0, and 461.5 μg/L, for the “low”, “medium”, and “high” intake groups, respectively; *p* = 0.002). For the comparison of between-group consumption of seafood, statistically significant difference was found for the group low vs high intake (*p* = 0.005), but not low vs medium intake (*p* = 0.07) and medium vs high intake (*p* = 0.33). There was a tendency for higher median UIC for those groups with more seaweed intake, but again it was not statistically significant. No tendency or significant difference in median UIC was observed for other food groups (Table 4).

**Table 4:** Urinary iodine concentration according to frequency of food type intake.

	Low	Medium	High	*p*-Value
Seaweed	212.3 (101.5–535.9) n = 174	250.6 (106.4–503.6) n = 68	318.0 (135.4–517.4) n = 15	0.58
Fish	209.4 (100.3–489.6) n = 100	261.1 (123.8–551.4) n = 108	214.6 (90.9–475.1) n = 49	0.51
Seafood	187.8 (98.1–448.5) n = 145	280.0 (132.3–634.1) n = 87	461.5 (241.9–585.5) n = 25	0.001
Dairy	242.2 (120.0–566.3) n = 47	251.2 (100.4–505.7) n = 60	223.0 (107.2–513.2), n = 150	0.86
Eat out	325.1 (105.5–555.2) n = 31	207.7 (98.8–524.2) n = 84	242.3 (113.9–482.4), n = 142	0.72

Low: Intake less than or equal to one day per week; medium: intake equals to three days per week; high: intake more than or equal to 5 days per week

## Discussion

We reported the iodine nutritional status of Taiwanese pregnant women who received prenatal checkups in a medical center in Northern Taiwan. The median UIC of the total surveyed women was 225.3 μg/L, indicating a sufficient iodine status according to the pregnancy values proposed by the WHO/UNICEF/IGN [17]. In non-pregnant women, the median UIC defining adequate iodine status is between 100–199 μg/L and the WHO/UNICEF/IGN also suggested that no more than 50% of the population should have a UIC < 100 μg/L, and no more than 20% of the population should have a UIC < 50 μg/L. In the current study, 17.1% of the total surveyed women was in the UIC range of 150–249 μg/L, and 35.4% of them had a UIC <150 μg/L, and 25.7% with a UIC ≥500 μg/L. However, there are no suggestions for distribution of UIC in pregnant women, making interpretation of the percentages challenging. Overall, the median UIC found in this study suggests adequate iodine nutrition in Taiwanese pregnant women but potential problems of both iodine deficiency and iodine excess may still be present.

In 1993, universal salt iodization (USI) was recommend by the WHO as the main strategy to eliminate IDD [24]. Nevertheless, instead of USI, the Taiwan government increased the iodine content in fortified salts on the basis of a voluntary salt iodization strategy [16]. In a 2012 salt survey in Taiwan, only 8 out of the 24 types of regular/plain salt available in the market were iodized [25]. The increasing amount of non-iodized salt in the market was believed to be one of the causes of the re-emergence of iodine deficiency in Taiwan in 2013 [15]. For populations that use non-iodized salt or use little salt for personal or health concerns (such as hypertension control), they won’t benefit from the policy of increasing iodine content of fortified salts and would remain iodine deficient. On the contrary, the above-mentioned policy may place some populations at risk for iodine excess if their iodine status is already at the high normal range under iodized salt use. The current study, conducted shortly after the policy implementation in 2017, may serve as an early warning sign for nutritional imbalance. More efforts to maintain iodine status within the optimal range is urgently needed. USI is still considered to be a better solution to improve iodine inadequacy [24].

Taipei Veterans General Hospital is a tertiary referral center located in the capital city of Taiwan. The population utilizing this hospital mainly resides in the Taipei metropolitan area and surrounding counties and generally are socioeconomically wealthier than the rest of Taiwan [26]. These people also receive longer education and tend to be pregnant at later ages of life [26]. The mean age at women’s delivery in this study was 33.89 years old, higher than the mean age of 31.97 for the whole Taiwanese population (according to birth registry of Taiwan 2017) [27]. The percentage of women with advanced maternal age (defined as age above 35 years old when pregnant) was also higher in our study (current study vs island-wide statistics in Taiwan: age 35 and above: 40.7% vs 29.4%, age 30–34: 43.2% vs 38.4%, and age 20–29: 16.7% vs 32.2%). Advanced maternal age has been shown to be associated with higher obstetrical complications [28]. Therefore, our participants may represent a group of pregnant women with higher obstetrical risk. However, health awareness is typically stronger in people with higher education and wealthier socioeconomic status [26]. This means that the studied women may have been on more nutritional support during pregnancy, evidenced by the data that prenatal multivitamin intake is highly prevalent in the studied women.

According to our post-study survey, 72.2% (n = 13/18) of various types of prenatal multivitamin available in the market in Taiwan contain iodine (S1 Fig). Among the 13 iodine-containing prenatal multivitamins, the iodine content ranges from 75 μg to 250 μg (median = 150 μg). The tendency for median UIC to be higher in the groups with prenatal multivitamin intake suggests that iodine supplementation may have also influenced the iodine nutritional status in the studied women. The lack of statistically significant difference may be related to the smaller case number. While iodine supplementation may be helpful in ensuring optimal iodine status in some countries, such as the U.S.A. and Canada (where 150 μg of iodine supplementation was routinely suggested during pregnancy and lactation) [29], caution should also be taken to avoid over-supplementation. For instance, taking a prenatal multivitamin containing 250 μg daily would be too much for someone who is already taking iodized salt and frequent dairy products. The median UIC of 241.5 and 242.6 μg/L in the groups taking prenatal multivitamin in this survey is at the edge of the upper limit for iodine adequacy. Further supplementation may tip the overall median UIC above the adequate range. Although statistically non-significant, the percentage of women with UIC ≥500 μg/L was higher in those groups with prenatal multivitamin intake. This warrants attention because iodine excess, as well as iodine deficiency, can lead to serious consequences [30–32].

For normal adults living in iodine-sufficient regions, their thyroid glands are subjective to physiologic adaptation to large amounts of iodine intake to prevent the occurrence of hyperthyroidism, namely the acute Wolff-Chaikoff effect [33]. However, some individuals may fail to escape from the acute Wolff-Chaikoff effect, rendering them susceptible to hypothyroidism under excessive iodine intake [34–36]. The ability to escape from the acute Wolff-Chaikoff effect does not mature until around 36 weeks of gestation, making the fetus especially vulnerable to the adverse effects of large amounts of iodine [37]. Long-term excessive iodine intake in pregnancy may lead to thyroid dysfunction, either hyper- or hypothyroidism [30, 38, 39]. Therefore, the American Thyroid Association (ATA) recently lowered the tolerable upper limit (UL) for iodine exposure in pregnancy to 500 μg per day, a level much less than the level of 1100 μg for adults in general [29]. The high percentage of women in the range of UIC ≥500 μg/L in this study is of particular concern after the ATA lowered the UL. To maintain iodine adequacy, nutritional strategies should be adjusted based on regional differences in iodine intake and iodine status.

The median UIC for women of child-bearing age in the stratum including participants living in the Taipei metropolitan area in NAHSIT 2013 was 143 μg/L, very different from the median UIC of 225.3 μg/L in this study [40]. The median UIC of a previous small-scale survey of pregnant women was around 128–144 μg/L [41]. Several aspects however should be considered when interpreting the discrepancy across studies. As mentioned earlier, salt labeling, increased iodine content in fortified salts took place in 2017, which may be part of the reasons for obtaining different results in 2013 and 2018. In addition, the high prevalence of prenatal multivitamin intake had also influenced the median UIC in this study. Last, emphasis on food nutrition during gestation may play a key role in maintaining iodine nutrition. The median UIC of 192.7 μg/L in the group that never took prenatal multivitamin in this study is suggestive of adequate iodine nutrition. This means that intake of dairy products, fish and seafood may have supported the iodine nutritional status in the studied women without multivitamin supplementation.

Overall, the median UIC level was higher in the early trimesters than in the late trimester in this study. A similar trend was reported in a Japanese study with decreasing median UIC from 221 μg/L in the first trimester to 193 μg/L in the third trimester [42]. In several areas in Iran, Switzerland, Australia, and Ireland, such a tendency has been repeatedly observed [43–46]. In the early trimester in pregnancy, increased glomerular filtration rate may lead to increased iodinuria and an overestimation of iodine nutrition [46]. In the late trimester in pregnancy, passage of iodine from the mother to the fetus increases and may cause decreased maternal urinary iodine excretion [47]. This decrease in UIC does not necessarily mean insufficient iodine pool in the mother [47]. However, to determine iodine adequacy in pregnancy, gestation-appropriate UIC ranges or standardized value for urine iodine/urine creatinine are needed [46]. Whether the median UIC continues to decrease after delivery should also be determined in future studies.

This study has several limitations. First, the findings observed in this study could only be representative of the pregnant women living in the metropolis or suburb in Northern Taiwan. Second, the results derived from subgroup analysis conducted in this study may be invalid due to the high variation in UIC in various subgroups attributable to the small sample size. Third, the non-validated FFQ employed in this survey is a simple questionnaire designed for the busy environment in the hospital setting. We therefore do not have information on the portions/servings of various foods that may contain iodine as well as the iodine content of individual foods. More detailed quantitative dietary investigation (i.e., 24hr dietary record) and more comprehensive databases on iodine content in foods and supplements are needed to better evaluate the association between UIC and dietary iodine intake. In addition, recall bias exists in all forms of questionnaire survey including this study. However, we believe the recall bias present in this study is likely to be non-differential and would bias the results toward the null value. Finally, because pregnancy is usually confirmed at 2 months and women may initially have a checkup at local clinics, participants who are in their first trimester is very limited in this study.

## Conclusions

Overall, our results indicate that the iodine nutritional status of pregnant women in the urban area of Northern Taiwan is adequate. However, efforts to eliminate iodine deficiency while avoiding iodine excess is still needed.

## Supporting information

S1 Appendix The food frequency questionnaire used in this study. (DOCX)

S1 Fig Prenatal multivitamin survey with iodine content in each brand of iodine-containing prenatal supplement. (PPTX)
