# Supplementary material for: Iodine nutritional status of pregnant women in an urban area of northern Taiwan in 2018
Source: PLoS One. 2020 May 15;15(5):e0233162. doi: 10.1371/journal.pone.0233162 (PMC7228086; doi:10.1371/journal.pone.0233162)
Supplement: S1 Appendix — (DOCX) [file pone.0233162.s001.docx]

**Iodine Nutrition Questionnaire**

Name: ___________ Birthday (year/month/day): ____________ date: _____________

Place of residence: ___________ city ___________ district

1. **How many days in a week do you eat a certain type of food during gestation?**
2. Seaweed: kelp, laver

- 7 days □ 5 days □ 3 days □ 1 day □ never

1. Fish

- 7 days □ 5 days □ 3 days □ 1 day □ never

1. Seafood (exclude fish)

- 7 days □ 5 days □ 3 days □ 1 day □ never

1. Dairy food: milk, cheese, yogurt, butter, ice cream

- 7 days □ 5 days □ 3 days □ 1 day □ never

1. Prenatal multivitamin

- 7 days □ 5 days □ 3 days □ 1 day □ never

1. **How often do you eat outside in a week during gestation?**

- 7 days □ 5 days □ 3 days □ 1 day □ never

1. **What kind of salt (iodized or non-iodized) do you use at home?**

□ iodized □ non-iodized □ I don’t know
