# Supplementary material for: Iodine nutritional status of pregnant women in an urban area of northern Taiwan in 2018
Source: PLoS One. 2020 May 15;15(5):e0233162. doi: 10.1371/journal.pone.0233162 (PMC7228086; doi:10.1371/journal.pone.0233162)
Supplement: S1 Fig — (PPTX) [file pone.0233162.s002.pptx]

## Slide 1
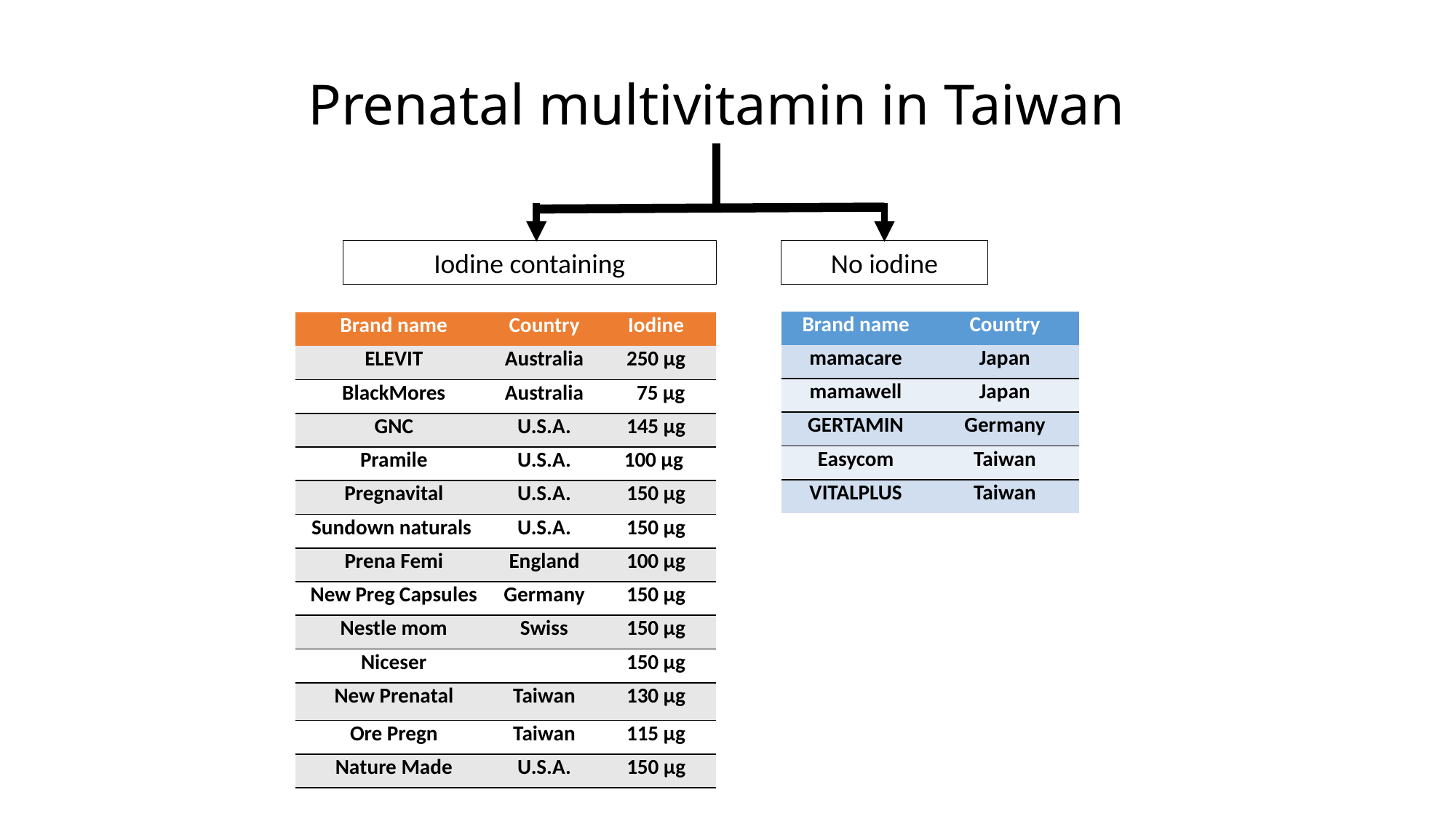

Prenatal multivitamin in Taiwan
Iodine containing
No iodine
| Brand name | Country |
| --- | --- |
| mamacare | Japan |
| mamawell | Japan |
| GERTAMIN | Germany |
| Easycom | Taiwan |
| VITALPLUS | Taiwan |
| Brand name | Country | Iodine |
| --- | --- | --- |
| ELEVIT | Australia | 250 µg |
| BlackMores | Australia | 75 µg |
| GNC | U.S.A. | 145 µg |
| Pramile | U.S.A. | 100 µg |
| Pregnavital | U.S.A. | 150 µg |
| Sundown naturals | U.S.A. | 150 µg |
| Prena Femi | England | 100 µg |
| New Preg Capsules | Germany | 150 µg |
| Nestle mom | Swiss | 150 µg |
| Niceser | | 150 µg |
| New Prenatal | Taiwan | 130 µg |
| Ore Pregn | Taiwan | 115 µg |
| Nature Made | U.S.A. | 150 µg |
| | | |
